# i The institutional review board is an impediment to human research: the result is more animal-based research

**DOI:** 10.1186/1747-5341-6-12

**Published:** 2011-06-07

**Authors:** Mark J Rice

**Affiliations:** 1 Department of Anesthesiology, Department of Anesthesiology, University of Florida College of Medicine, PO Box 100254, Gainesville, FL 32610-0254, USA

## Abstract

Biomedical research today can be generally classified as human-based or nonhuman animal-based, each with separate and distinct review boards that must approve research protocols. Researchers wishing to work with humans or human tissues have become frustrated by the required burdensome approval panel, the Institutional Review Board. However, scientists have found it is much easier to work with the animal-based research review board, the Institutional Animal Care and Use Committee. Consequently, animals are used for investigations even when scientists believe these studies should be performed with humans or human tissue. This situation deserves attention from society and more specifically the animal protection and patient advocate communities, as neither patients nor animals are well served by the present situation.

## Background

This article seeks to explore and bind together four fundamental concepts: 1. Human-based research has been neglected in favor of animal-based research. 2. Human-based research offers clear benefits compared to animal-based research. 3. Physician-scientists believe that Institutional Review Boards (IRBs) are more difficult to deal with than Institutional Animal Care and Use Committees (IACUCs). 4. The difficulty in dealing with IRBs force many physician-scientists, who prefer human research, to perform research with animals.

Numerous groups have endorsed the reduction or elimination of nonhuman animals (hereafter referred to as animals) from research, but judging by the best estimates, this has not happened [1-3]. Indeed, the number of animals used in research is skyrocketing. Although the exact number of animals used per year in the USA have never been available, estimates for use in 1980 were approximately 20 million [4]. Estimates now approximate a half-billion [1,3] with genetically-modified animals counting for the majority. Clearly, efforts to reduce the total number of animals used in research have failed. This failure has been explored by others [5-10]. The explanations include, among others, tradition, institutional inertia, the large amounts of money involved in the process, the fact that it is statistically more likely to get animal-based research funded by the National Institutes of Health and other funding agencies than human-based research, overall lack of societal concern for animals, and animals in labs offer the researcher better control over variables. I will introduce what I believe to be the first essay proposing a regulatory-based explanation for the continued use of animal models in university-based research in spite of evidence that they may not be good models for human disease: regulatory burden.

Greek and Greek's recent article in this journal [7] brought new attention to a 1986 National Academy Press publication [11] that made public the fact that 50% or more of all extramural NIH funding is directed toward animal-based research. This is consistent with more recent publications [12-18]. It is estimated that roughly 70% of the NIH research budget goes to basic science [12,13] (which is animal intensive) and that the percentage in the UK is approximately the same [14-18]. In 2003, Clinical Research Roundtable (CCR) at the

Institute of Medicine published a report in the *Journal of the American Medical Association *stating that there is a "disconnection between the promise of basic science and the delivery of better health" [16]. The CRR also pointed out that clinical research received about half the money that basic science received [16], which is consistent with the 70% figure for basic research funding cited above.

Moreover, the amount of basic research being translated into human treatments appears to be at an all time low [19-23]. An editorial in *Nature *[24] lamented the fact that every week the scientific community hears of animals being cured of some disease, but these advances are not translating to humans. The pharmaceutical industry is also developing fewer new chemical entities (NCEs) to test in the clinics [25].

I acknowledge that the use of animals in research is contentious issue, both scientifically [2,5,8,9,26-29] and ethically [30-39] with positions ranging from the animal rightist Tom Regan to the equally adamant animal-based research defender Carl Cohen. However, I am not an animal protectionist, welfarist, or rightist. I am a physician-scientist, who prefers to do human clinical research. I have migrated to this position, in part, because I now question if animal-based research *per se *is predictive for the human being modeled [2,8]. (See below for more on this.) I am writing this article because I believe the animal protection and patient advocacy communities are neglecting an area which, if reformed, would help animals, humans, and researchers alike.

I spent the early part of my career as a physician-scientist in the laboratory that developed the solution that is now used throughout the world to preserve solid organs for transplantation [40-44]. This was rewarding work, resulting in saving thousands of lives because of the extension of organ viability prior to transplantation. Many animals were used in this process and much was learned from their use. For a number of reasons, as my career progressed, I became more interested in doing human clinical research. One reason was my disillusionment with using animals to model human conditions. Sometimes, animal models worked very well but many times they did not--the problem was that prospectively we didn't know into which category the laboratory work would fall. It seemed my time, effort, and valuable resources could be better spent working directly with humans.

My concerns have been substantiated in the scientific literature. Spanhaak et al. analyzed Medline abstracts and European Public Assessment Reports, published by the European Medicines Agency (EMEA) to assess the ability of animals to predict hepatotoxicity. They reported a false negative rate of 269 out of 710 (38%) compounds based on Medline data and 70 out of 137 (51%) based on EMEA data [45]. Johnson et al. discovered that out of 39 antineoplastic drugs tested on xenograft mice, only one mimicked the response in humans [46]. Drugs used in anesthesia are known to affect humans and animals differently [47]. The denial of the effects of smoking [48] and asbestos [49] were founded on studies in animals. Many studies and commentaries have bemoaned the fact that animal models cannot predict human response [50]. (I will address this more thoroughly below and for a much more thorough examination of this issue see [2,7,8].)

Our group has recently been successful in several research areas including our recent debunking of a myth regarding the anatomical nomenclature of the airway [51]. In 2003, Smith and colleagues [52], using magnetic resonance (MR) imaging, published a paper in one of our leading journals stating that a long-held practice in anesthesiology was unnecessary, and in fact possibly dangerous. Cricoid pressure is used during the induction of anesthesia by pressing on the airway, just below the thyroid cartilage on the cricoid ring, to compress the alimentary canal, preventing stomach contents from entering the mouth and possibly the airway to the lungs. This dreaded complication of anesthesia (aspiration) is frequently fatal. Smith stated that the alimentary canal at the level of the cricoid is the esophagus and they attempted to show, through a series of MR images, that the esophagus moved to the side during cricoid pressure and in fact did not protect the airway during this maneuver.

Our group, with one of the world's experts in neck anatomy as a coauthor, proved that the esophagus *does not even exist *at the level of the cricoid. The alimentary canal at this level is the post-cricoid hypopharynx, which is important because this structure is fixed with respect to the cricoid and does not move. The problem with the previous Smith study was: 1. That the cricoid is superior to the esophagus; thus pressure on it is not intended to close off the esophagus, but also; 2. The cricoid pressure unit is at the level of the cricoid and is attached to other structures and thus, when pressure is applied, this pressure translates straight down and the alimentary canal can be closed off to prevent GI contents going into the trachea and lungs.

This study was done in humans because we were interested in the anatomy of ... humans. In addition, the airways of the animals most commonly used in laboratories are not the same as the human airway [53-62] and we believed animal imaging would have been of no assistance in proving our hypothesis. While the above is certainly anecdotal, the work referenced below confirms my opinion that human-based research is superior to animal-based research when the goal is diagnosis and treatment of human disease.

## Animals in Research

I have no ethical inhibition about using animals to find cures for human disease. The current law allows using animals in research and regulates the process; my concern for their well-being stops there. Furthermore, there is no doubt that animals can be successfully used for basic science research and I support such uses. For example, animals can be, and historically have been, used as heuristic devices, in basic research, as bioreactors, to teach surgical procedures and so forth [2]. But using them as predictive models or what has been called *causal analogical models *[9], in applied research, such as drug and disease research specifically, has not been a useful exercise [2,8].

For example, in a *Nature Medicine *editorial introduction to two articles, one by by Van Dyke [63] and another by Ellis and Fidler [64], the editorial stated: "The complexity of human metastatic cancer is difficult to mimic in mouse models. As a consequence, seemingly successful studies in murine models do not translate into success in late phases of clinical trials, pouring money, time and people's hope down the drain" [64]. Ellis and Fidler stated: "Preclinical models, unfortunately, seldom reflect the disease state within humans ..." [64]

One reason for the high cost of medications today is the fact that drugs fail late in development. Only about 11% of all drugs entering Phase I human clinical trials make it to the market (the failure rate for cancer drugs is around 95%) [65]. Paul stated: "The higher failure rates in these areas [cancer and drugs acting on the CNS] are in part due to the relatively unprecedented nature of the drug targets being pursued and to the lack of animal models with a strong capacity to predict human efficacy" [66]. A majority of drugs that enter Phase III also fail. These failures are caused in large part by the failures of the preclinical animal modes to predict human responses[67-75]. The FDA has also acknowledged this [76]. The other oft-overlooked factor is that animal models have misled scientists into taking wrong research paths and have perhaps kept treatments off the market [77]. In my own field of anesthesiology there are distinct differences in drug response among species [47]. Furthermore, animal testing has failed to prevent tragedies like the death of gene transplant recipient Jesse Gelsinger [78] and the morbidity of the six volunteers testing TGN1412 [71,79,80]. Wenner: "Wilson and the rest of the scientific community had to learn the hard way "that what you've learned from animals will not necessarily predict what's going to happen in humans"" [78].

The animal model has been equally misleading in research into mechanisms of diseases such as Alzheimer's [81-83], mesothelioma [84-86], smoking-induced cancer [48,87], cancers in general [88,50,77], stroke [89-91], HIV/AIDS [92-99] and others [100]. Even transgenic animal models have been disappointing [101,102]. A promising but greatly underfunded area of research involves studying humans who, despite repeated exposure to HIV, do not become infected [103].

One reason for the above failures is that humans vary considerably in their response to drugs and even diseases [104-113], thus expecting a different species to predict human response is naïve. The question thus arises: *Why do scientists use animals when so much research exists that can and should be performed with humans or human tissue?*

The goal of virtually all biomedical research is human applications and it makes sense to study *Homo sapiens*. Grant et al. noted that the "United Kingdom spends over £1600 million a year on non-commercial biomedical and health services research. The tacit understanding is that the biomedical research these bodies support will lead to an eventual improvement in health" [114]. Dorsey et al. [115] and Boat [116] both pointed out in 2010 that progress in biomedical research must be judged by gains in the health of the human population. This tacit understanding has implications for what sort of research is funded. All good science is not *ipso facto *going to advance medical care.

**Figure 1:**
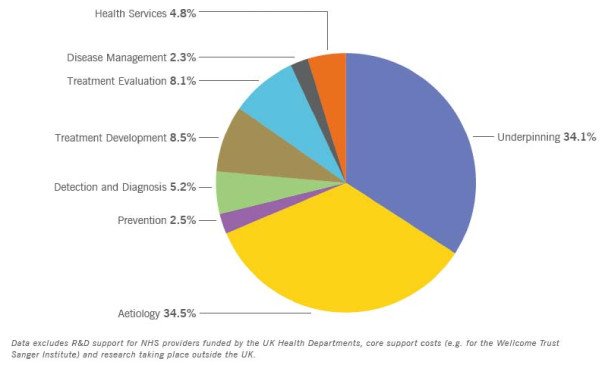
**UK Health Research Analysis**. Proportion of Combined Total Spend by Research Activity

Rothwell [84] pointed out that studying humans and the physical sciences have been the most productive compared to animal research. He began by explaining that 90% of non-National Health Service Research & Development and non-industry funding in the UK came from the Medical Research Council and three other charities (see table 1[117] and figure 1[118]). Of this funding, a very small percentage was spent on patient-oriented clinical research. For example, the Wellcome Trust spent less than 3.4% on diagnosis and treatment evaluation. Others have also criticized the proportion of funds spent on nonclinical research [119,120]. Rothwell also condemned the British system for promoting basic scientists to clinical chairs. But, breakthroughs do not necessarily come from the target of resources. Rothwell:

**Table 1:** Research expenditure of research funds by area of research activity

Research Activity	Percentage of research funding			
	
	MRC	Wellcome	BHF	CRUK
Underpinning*^†^	41.2	49.2	27.5	24.3

Aetiology^†^	38.5	40.5	48.8	35.2

Prevention	2.9	1.9	1.8	2.1

Detection and diagnosis	4.5	1.7	6.0	6.1

Treatment development^†^	5.6	4.3	9.3	17.3

Disease management	4.5	1.7	5.2	11.7

Health services	1.6	0.5	0.6	0.4

MRC = Medical Research Council, BHF = British Heart Foundation, CRUK = Cancer Research UK. *Research aimed at understanding normal biological development and functioning. ^†^Mostly laboratory-based.

Indeed, most major therapeutic developments over the past few decades have been due to simple clinical innovation coupled with advances in physics and engineering rather than to laboratory-based medical research. The clinical benefits of advances in surgery, for example, such as joint replacement, cataract removal, endoscopic treatment of gastrointestinal or urological disease, endovascular interventions (e.g., coronary and peripheral angioplasty/stenting or coiling of cerebral aneurysms), minimally invasive surgery, and stereotactic neurosurgery, to name but a few, have been incalculable. Yet only a fraction of non-industry research funding has been targeted at such clinical innovation. How much more might otherwise have been achieved? [117]

Other studies support this opinion [119-123].

Rothwell goes on to point out that much of the failure of basic research can be attributed to the use of animal models. Ledford continues this theme in discussing how Nobel laureate Sydney Brenner brought an audience at the American Association for Cancer Research to its feet when he stated that *Homo sapiens *should be the model organism for disease research. This is especially noteworthy as Brenner won the Nobel Prize for his work on *Caenorhabditis elegans *[124].

Additionally, there are risks to using animals in basic research compared to studying humans in the clinical arena. Alini et al. expressed concern that the use of some animals models "... serve to spread incorrect information about the processes involved in disc degeneration and about the possibilities of repair" [125]. They continue:

If we want to improve understanding of pathology and treatment of human IVDs [intervertebral discs], legislation should change to allow us easier access to human tissues, e.g. from pathological, cadaveric and organ donor source. The fundamental question to be addressed is: *is it more (un)ethical to use animal models, which we know do not represent any human disc pathological conditions, or to use human tissues with all the ethical issues? We collectively believe the answer is clear: human tissue needs to be made more available! *[125] (Emphasis added.)

Why isn't there more human tissue available for study? I believe there is a critically important reason.

## Protocol approval from local review boards: IACUC and IRB

I want to contrast the ease of working through the regulatory framework for animal-based research, (approval from the local IACUC), with the difficulty of the parallel regulation involved in clinical research (approval from the local IRB). I believe this is one major reason scientists choose to work with animals instead of performing clinical research--the regulatory hurdle is simply much lower.

Plous and Herzog [126] upset many in the scientific community with a 2001 study published in *Science*. They submitted protocols from one institution's IACUC to the IACUCs of a number of different institutions and found that "regardless of whether the research involved terminal or painful procedures, IACUC protocol reviews did not exceed chance levels of intercommittee agreement." Almost all protocols were approved at the local IACUC, but were approved at other IACUCs only a small percentage of the time. In other words, local IACUCs favored the home research applicants, with very easy protocol approval. They also showed that even though almost all animal studies in their sample were approved locally, the IACUC reviewers thought that only 45% of these submissions were either good or excellent. In addition, approximately 35% of the submissions were either mostly not understood or not understood at all. Finally, they deemed that 31% were either not very valuable or not valuable at all. According to the Plous study, IACUC approval is easy to obtain.

Perhaps Carbone, a veterinarian and long member of an IACUC, since these were mandated in 1986, summed up the role of the IACUC best when he stated that IACUCs do not "function by rejecting animal protocols" and that "this is especially true if a project has been favorably peer-reviewed by a competitive granting agency such as the NIH." He concludes by stating that "the current nature of animal protocol review, [is one] in which virtually any research procedure may be approved so long as it is justified by its scientific value" [127] p183-4. I am not insinuating that the IACUC approves everything without revisions or that they unthinkingly rubberstamp all applications. But in the final analysis, the result is that a vast majority of everything submitted ultimately gains approval and in a timely fashion after the usual modifications.

In contrast to working with IACUCs, it is the opinion of many physician-scientists that protocol approval from IRBs is very difficult. Allow me to note that IRBs *should *conscientiously and diligently protect human participants in research. The following are studies showing just how costly--both in terms of time and money-- human-based research is and how difficult the navigation through the IRB process can be for researchers.

The Infectious Disease Society of America [128] published a damning manuscript in 2009, outlining the tremendous burden of current regulations on the progression of human clinical research. They reported that local review by IRBs of multi-center studies does not improve either protocols or informed consents. Greene and Geiger studied multiple IRB reviews of multicenter clinical trials and observational studies. They reported a total of 40 peer-review articles in addition to six reports from key sources. They found vastly different requirements across various IRBs [129]. Ravina et al "examined the costs and effects of local IRB review of the consent and protocol in a multicenter clinical trial in Parkinson disease." The found that "Seventy-six percent of changes to the consent [form] reflected standard institutional language, with no substantive changes to the protocol. The costs of this process exceeded $100,000" [130].

Schneider et al. found that despite researchers' use of an IRB protocol and consent previously approved by Harvard and Rand IRBs and the National Cancer Institute's IRB, the IRB in 20 out of 65 institutions participating in a quality of care cancer study required changes to the protocol resulting in time delays of more than one year. Thirty-five required modification to the consent form. Six IRBs required the researcher's to obtain active consent from the attending physician prior to contacting the patent, which severely limited patient recruitment [131]. Finch et al. studied 75 IRBs and found studies associated with local IRBs had lower participation rates and more effort was required to navigate the process [132]. Green et al. reviewed IRBs for variation on observational health services research and concluded:

Several features of the IRB system as currently configured impose costly burdens of administrative activity and delay on observational health services research studies, and paradoxically decrease protection of human subjects. Central review with local opt-out, cooperative review, or a system of peer review could reduce costs and improve protection of human subjects[133].

The above themes have been echoed in numerous other studies [129,134-141].

Inter-IRB variability has been well established and studies of individual IRBs also suggest that they require changes that are arbitrary. It appears that very little is being realized despite a process that is very resource consuming in terms of patient participation, time, and money. Promotions in academia (at least in the USA) are based on the number of papers published. One cannot publish clinically-based research while waiting for the local IRB to approve a protocol. It is my experience that one can receive IACUC approval and accomplish a great deal of research in the time it takes to get a single protocol IRB-approved. But, not only is the IRB process very time-consuming, it is also very costly [130]. Dollars that could be spent on research are unnecessarily channeled into IRB preparation cost and these expenditures almost never improve the research. Anecdotally, our department has hired a professional just to handle IRB submissions because of their difficulty. No such position is required for IACUC submissions.

It is clearly necessary to have close oversight of human clinical trials. The errors of days past, when research subjects were abused, can never be repeated. I believe that the current IACUC review system results in sometimes-lax oversight, while the current IRB is an absolute hindrance to medical advancement. As this paper is not about IACUC regulation per se I will not address changes that might be appropriate for IACUCs. I leave for others the challenge to steer animal-based research protocols between the overly burdensome, irrelevant, and adversarial regulation and the cozy old buddy systems that merely rubber-stamps. However, the incredibly burdensome IRB system, bogged down by document minutia, has not been shown to be effective. I am not advocating research without accountability. In fact, I recently authored an opinion piece calling for the naming an archiving author, whose responsibility it is to keep all original research data for ten years following publication [142]. This editorial, which is the first of its kind, was partially in response to academic misconduct, most famously portrayed by the Ruben anesthesia misconduct scandal [143,144]. (The irony is that Ruben conducted several studies without IRB approval, although he stated he received approval in the publications. The IRB process did not protect patient welfare as it was intended.)

The situation as it exists, however, is causing scientists to use animals in research rather than work with human models because of the large discrepancy in regulatory ease between the two types of research. Based on discussions I have had with scientists in my university and other universities where I have been a visiting professor and or collaborator with other scientists, this problem is real and is pushing many academics away from human research. I realize that I have presented no hard evidence that quantifies this problem. However, my hope is that such studies will be done in the future and believe that this article will stimulate such research. But the fact that no such studies currently exist should not be taken as proof my position is false. In medicine, case reports have historically been used as harbingers of a brewing problem on the horizon. My position is based on communication with many scientists, in many different institutions (personal communication) and is supported by studies examining overlapping areas.

Another personal note: I am currently involved in evaluating a potential blood coagulation product. The advice from established local researchers was to test the concept *in vivo *in rabbits, then in dogs. As the product is designed to test blood for certain properties, I suggested that we study human blood in a much more relevant *in vitro *model, avoiding many of the known and unknown animal blood interferences. The knee-jerk reaction is to test with an animal model even when testing on human tissue (like blood) would be easier and clearly more clinically applicable.

## Human-based research

The above must be considered in light of the fact that research and the practice of medicine are now focusing on the differences between individual humans [106,107,109,145-158], not the commonalities between humans and other animals. Furthermore, NIH and other granting bodies are actively pushing translational research. But even translational research, when based on animal studies, has been problematic. Höerig and Pullman cautioned that translational research is based on the premise that *in vivo *animals studies can be translated to humans but that "animal models themselves have a poor record of predicting human disease outcome ..." [159]

Some believe the basis for beginning first-line research should be human observation, not animal studies. Marincola [160] noted that the scientific establishment currently favors research in the form of hypothesis testing but ignores the fact that good hypotheses come from the observation of humans. Without a good hypothesis to test, the entire exercise becomes suspect. Marincola goes on to describe why human observation should be held in higher esteem and should not be dismissed as "just descriptive." He also notes: "For example, in animal models, Interleukin-23 can either promote or hamper cancer growth; yet, information about its bio-availability in human cancers and its modality of expression, information that can potentially provide insight into the interpretation of such models, is limited" [160].

There appears to be a certain blindness to this problem and a frantic effort to find explanations for the animal-model failure. Ioannidis presents a good example of this mindset:

There is considerable evidence that the translation rate of major basic science promises to clinical applications has been inefficient and disappointing. The deficiencies of translational science have often been proposed as an explanation for this failure. An alternative explanation is that until recently basic science advances have made oversimplified assumptions that have not matched the true etiological complexity of most common diseases... [161]

As Ioannidis states, "Even the most promising findings of basic research take a long time to translate into clinical experimentation, and adoption in clinical practice is rare" [19]. Jin and Wang echo this when they point out that pathologies in humans represent complex genetic diversity while animal models are usually inbred strains. They also correctly point out that treatments developed in animals rarely succeed in humans [162].

## Conclusion

This paper has tied together the following four concepts: 1. Human-based research has been neglected in favor of animal-based research. 2. Human-based research offers clear benefits compared to animal-based research. 3. Physician-scientists believe that Institutional Review Boards (IRBs) are more difficult to deal with than Institutional Animal Care and Use Committees (IACUCs) and available evidence supports this. 4. The difficulty in dealing with IRBs forces many physician-scientists, who prefer human research, to perform research with animals.

I believe this is the first essay proposing a regulatory-based explanation for the continued use of animal models in university-based research in spite of evidence that they may not be good models for human disease. Research has shown that local IACUCs approve essentially everything they consider. This is not to say IACUCs are a rubber stamp, rather that the scientist working with them can be confident of eventual approval. While there is variation among IACUCs, a scientist applying for permission for animal-based research can be more confident than the scientist applying through the local IRB that the protocol will be approved. This has implications for scientists in the initial stage of their career must decide between pursuing a career that involves human-based research or animal-based. It also has implications for experienced scientists who tire of the bureaucratic process that involves IRBs. Scientists who need to attract funding for their research will naturally be attracted to the path of least resistance in order to receive approval for their medical research projects. The much easier approval of IACUC compared to IRB protocols offers a number of obvious advantages including bringing money into the university as well as more publications per year - the most important key to academic promotion. The variation among IACUCs per Plous and Herzog is probably similar to that among IRBs (more research is needed). However, the implications for this differ. The human-based researcher may need to deal with multiple IRBs simultaneously during multicenter trials while the animal-based researcher has only the local IACUC to consider. In addition, the IRB appears to be far less efficient, more costly, and imposes a more intricate, arbitrary, and difficult regulatory burden even if the scientist is only dealing with his local IRB.

I acknowledge that much more research needs to be performed in order to provide adequate evidence to justify my conclusions. This is but an introductory paper, not be the final word on anything, as I have obviously not answered most let alone all the questions. A PubMed search for ""Institutional Animal Care and Use Committee" and "Institutional Review Board"" revealed four papers, none of which were relevant to this topic. Studies are needed that survey those in the scientific community who have worked with both the IRB and the IACUC asking specifically about the comparative difficulty of working with each. Research should also be conducted comparing the length of time and number of changes required between the two review panels. Comparison could be made between the first application and final approved application. The applications could then be submitted to third party review panels for review and judging as was done in the Plous study [126]. Surveys could be performed among young physicians contemplating research careers in an attempt to ascertain why the physicians choose to study animals or humans. Follow up surveys could also be attempted five years later to see how the plan played out. Nevertheless, this introduction offers a reasonable, testable and novel explanation for current thinking among physician-scientists.

As long as animal-based studies are funded to the neglect of human-based studies and animal-based studies reward the research institution with high overhead costs, there will be pressure to migrate toward animal studies. In order for a long-term satisfactory solution to be reached, the funding process as a whole must be addressed. That being said, we should not wait for a perfect solution before implementing a better system. Standardizing IRBs would be such a step in the right direction. The National Cancer Institute's (NCI's) Central Institutional Review Board (CIRB) was studied by Wagner et al. This board functions as a central review board to conduct a single review for the NCI's multisite phase III oncology trials. They found that "CIRB affiliation was associated with faster reviews ... fewer hours of research staff effort ... a savings of [money]" [163].

In the final analysis, society must voice its support for safe human-based research. This outcome can be facilitated by physician-scientists speaking out on the importance of our research both in the scientific literature and in public forums. Humans volunteering to participate in clinical research deserve the best protection available. However, IRBs have become so onerous that physician-scientists are being forced to rethink human research. In contrast, it has been shown that IACUCs approve essentially all local protocols. Neither situation is optimal, but if a scientist is contemplating research with humans or with animals, the disparity in regulatory bodies has and will continue to influence the decision-making process. Animal protectionists seeking to redirect scientists from animals-based research to human-based research should become involved with their local IRB. There is a universal requirement that local leaders, from all walks of life, join local IRBs. Animal protectionists can certainly participate on these boards and as a member of the local community, contribute to their functioning. This essay has presented one more important reason to participate.

## Competing interests

The authors declare that they have no competing interests.

## About the author

Mark Rice, MD has been on faculty in the Departments of Anesthesiology at the University of Wisconsin-Madison and the University of Florida (UF). He is chief of the liver transplant division at UF Department of Anesthesiology, has seven US patents, and reviews for several major journals.
